# Retraction: Downregulated FOXO3a associates with poor prognosis and promotes cell invasion and migration via WNT/β-catenin signaling in cervical carcinoma

**DOI:** 10.3389/fonc.2026.1961022

**Published:** 2026-08-18

**Authors:** Frontiers Editorial Office

The journal retracts the 01 October 2019 article cited above.

Following publication, concerns were raised regarding the integrity of the images in the published figures. Image duplication concerns were identified in Figures 3–5 and 7.

The authors failed to provide a satisfactory explanation during the investigation, which was conducted in accordance with Frontiers’ policies. As a result, the data and conclusions of the article have been deemed unreliable and the article has been retracted.

This retraction was approved by the Chief Editors of Frontiers in Oncology and the Chief Executive Editor of Frontiers. The authors agree to this retraction.

Frontiers would like to thank Sholto David for contacting the journal regarding the published article.

